# Ductile-to-Brittle Transition and Brittle Fracture Stress of Ultrafine-Grained Low-Carbon Steel

**DOI:** 10.3390/ma14071634

**Published:** 2021-03-26

**Authors:** Tadanobu Inoue, Hai Qiu, Rintaro Ueji, Yuuji Kimura

**Affiliations:** National Institute for Materials Science, 1-2-1, Sengen, Tsukuba 305-0047, Japan; QIU.Hai@nims.go.jp (H.Q.); UEJI.Rintaro@nims.go.jp (R.U.); KIMURA.Yuuji@nims.go.jp (Y.K.)

**Keywords:** brittle fracture stress, fracture test, low-carbon steels, ultrafine grained microstructure, finite element analysis

## Abstract

Ductile-to-brittle transition (DBT) temperature and brittle fracture stress, *σ_F_*, are important toughness criteria for structural materials. In this paper, low-carbon steels with an ultrafine elongated grain (UFEG) structure (transverse grain size 1.2 μm) and with two ferrite (*α*)-pearlite structure with grain sizes 10 µm and 18 µm were prepared. The UFEG steel was fabricated using multipass warm biaxial rolling. The tensile tests with a cylindrical specimen and three-point bending tests with a single-edge-notched specimen were performed at −196 °C. The local stress near the notch was quantitatively calculated via finite element analysis (FEA). The *σ_F_* for each sample was quantified based on the experimental results and FEA. The relationship between *σ_F_* and *d_α_* in the wide range of 1.0 μm to 138 μm was plotted, including data from past literature. Finally, the conditions of grain size and temperature that cause DBT fracture in low-carbon steel were shown via the stress−*d*^−1/2^ map. The results quantitatively showed the superiority of *α* grain size for brittle fracture.

## 1. Introduction

Low-carbon steels are still the steel grades used in wide application due to their low price and good weldability, formability, and recyclability. They are normally composed of a ferrite(*α*)-pearlite microstructure through a thermomechanical control process [1,2], and they are changed to a *α*-cementite microstructure by refining *α* grains through a plastic deformation and heat treatment [3,4,5]. It is well known that yield strength, *σ_y_*, increases with a decrease in grain size, based on the Hall–Petch relationship [6,7,8,9]. Additionally, in uniaxial tensile tests, there are reports that the elongation is reduced by grain refinement, but the reduction in area is improved [10,11]. That is, ductility is not always deteriorated by grain refinement. With regard to toughness, which has a strong correlation with ductility, the ductile-to-brittle transition temperature (DBTT) obtained in the Charpy impact test is improved by refining the *α* grains [4,7,8]. This improvement in DBTT can be explained by the stress theory that the brittle fracture stress, *σ_F_*, increases significantly compared to the increase in *σ_y_* due to the refinement of *α* grains [4,12,13]. The improvement in *σ_F_* is one of the advantages of grain refinement. However, the *σ_F_* in ultrafine-grained steels has been little reported quantitatively, compared with the mechanical properties of *σ_y_*, ductility, DBTT, etc. This is attributed to the fact that steels with an ultrafine-grained (UFG) structure have a strong texture depending on process, so it is difficult to produce a bulk sample with an ultrafine-grained (UFG) structure and the local stress cannot be obtained directly from experiments alone, unlike the mechanical properties [14].

In the present study, an 800 MPa class low-carbon steel with an ultrafine elongated grain (UFEG) structure with an average transverse grain size of 1.2 μm was created by warm biaxial rolling process. For comparison, two low-carbon steels with a *α*-pearlite structure with grain sizes 10 μm and 18 μm were also prepared. The three-point bending tests were performed at −196 °C, and the *σ_F_* near the notch tip was quantitatively estimated based on finite element analysis (FEA) and the experimental results. The relationship between *σ_F_* and *α* grain size in the range of 1.2 μm to 18 μm was plotted and compared with the data in the past literature [14,15,16] with *α* grain size in the range of 23 μm to 138 μm. Finally, in low-carbon steel, the conditions of the *α* grain size and temperatures that cause the ductile-to-brittle transition fracture were clarified via the stress-(grain size)^−1/2^ map.

## 2. Experimental Procedure

### 2.1. Specimen Preparation

**Figure 1:**
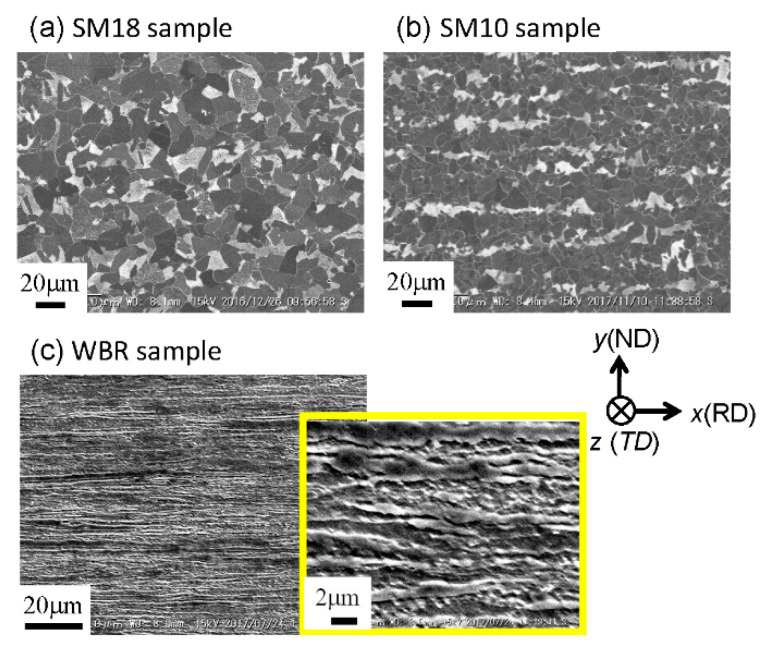
SEM images of the microstructures at the center of the cross-sectional plane parallel to the rolling direction (RD).

**Figure 2:**
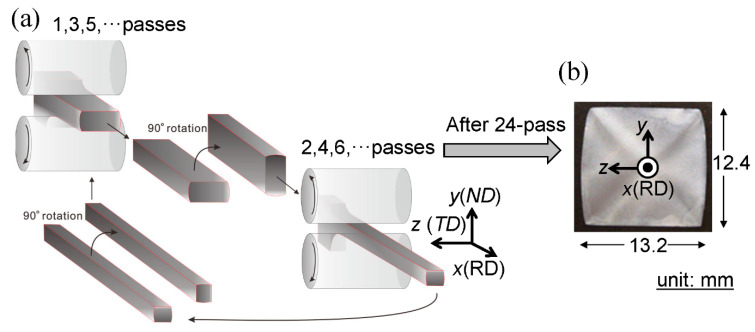
(**a**) Schematic illustration of the warm biaxial rolling (WBR) process and (**b**) a 13 mm square bar produced.

A low-carbon steel (0.15%C-0.3%Si-1.5%Mn) was used in this study. An ingot was prepared by vacuum melting and casting, homogenized at 1200 °C, hot-rolled to a 4 cm square bar by a caliber-rolling simulator (Oono-roll Corporation, Tokyo, Japan) [17], and then cut to 110 mm in length. The bar was hot-rolled to form a square bar with a cross-sectional area of 2 cm^2^ by the rolling simulator, then soaked to 900 °C for 1 h, followed by air cooling (SM18 sample). The Vickers hardness was 146 ± 4HV0.1. In order to create more finely grained structures, the hot-rolled 4 cm square bar was soaked to 900 °C for 1 h, and then it was caliber-rolled to form a square bar with a cross-sectional area of 2 cm^2^, followed by air cooling (SM10 sample). The hardness was 147 ± 9HV0.1. The scanning electron microscope (SEM), KEYWNCE VE-7800 (Keyence Corporation, Osaka, Japan) images of the microstructure for the SM18 and SM10 samples are shown in Figure 1a,b. Both samples have a typical *α*-pearlite structure. The average size of the *α* grain, *d_α_*, was approximately 18 μm for the SM18 sample and approximately 10 μm for the SM10 sample. Next, to obtain an UFG structure, the 4 cm square bar was austenitized at 900 °C for 1 h followed by ice-brine quenching. The UFG steel bar was produced via multipass biaxial rolling (WBR), as shown in Figure 2a, at a warm temperature of 550 °C. Eventually, a 13 mm square bar (WBR sample), shown in Figure 2b, was created. Detail of the WBR process was given previously [18,19]. The hardness was = 277 ± 7HV0.1. Figure 1c shows SEM images of the microstructure. The average transverse size, *d_t_*, of the *α* grains was 1.2 μm [18], which was the same size from 1/2t to 1/8t.

### 2.2. Mechanical Properties and Microstructure

**Figure 3:**
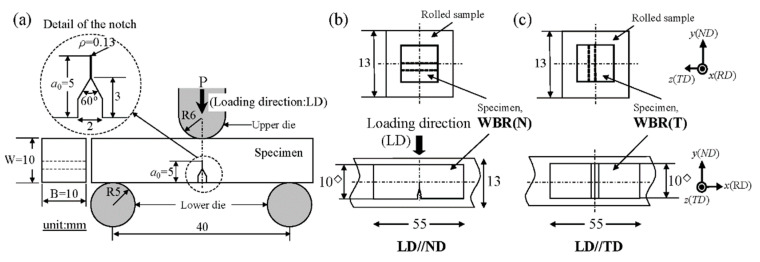
(**a**) Detail of the three-point bending test. (**b**,**c**) Notch orientation and dimensions of the specimens taken from the WBR sample.

Tensile tests at −196 °C were performed with a crosshead speed of 0.85 mm·min^−1^ using specimens with a round cross section of 6.0 mm and a gage length of 30 mm [20]. In order to obtain strength data on the anisotropy in the WBR sample for FEA, tensile tests of small plate specimens were also performed at −196 °C with a crosshead speed of 0.11 mm·min^−1^. The specimens with a parallel length of 2.6 mm, a width of 2 mm, and a thickness of 1 mm were machined from the bars in the RD, in the normal direction (ND), and in the transverse direction (TD) A three-point bending test at a crosshead speed of 0.5 mm min^−1^ was performed at −196 °C with a single-edge-notched specimen, as shown in Figure 3a. To verify the effect of the root radius (*ρ*) in initial notch on the *σ_F_*, the SM10 specimen with a notch of *ρ* = 0.25 mm was also prepared. For the WBR sample, in order to investigate the effect of notch orientation, two kinds of specimens, LD//ND (WBR(N)) and LD//TD (WBR(T)), were prepared, as shown in Figure 3b,c. The specimens after test were observed through a digital camera and a digital microscope, and the fracture surfaces were observed through SEM operated at 15kV. Electron backscattered diffraction (EBSD) in a Schottky-type SEM operated at 15kV was used for observing the microstructures (JEOL JSM-7001F, Tokyo, Japan). EBSD analysis was performed via a JEOL-7001F equipped with a TSL-OIM analytical system (TSL solutions, Sagamihara, Japan).

## 3. Numerical Procedure

**Figure 4:**
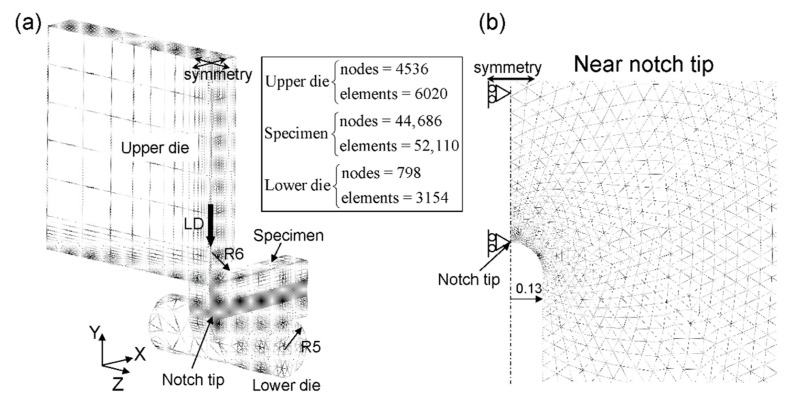
(**a**) Schematic illustration of the three-point bending test simulation and (**b**) the finite element mesh used for the specimen with an initial notch of *ρ* = 0.13 mm.

A three-dimensional elastic-plastic FEA was performed using the FE-code ABAQUS/Standard (ver.6.5.4, Dassault systems, Tokyo, Japan). A 1/4 model was used by adopting the symmetry condition, as shown in Figure 4a. A 20-node quadratic element was used for the specimen, and the mesh in the specimen included 44,686 nodes and 52,110 elements. An 8-node linear element was used for the upper die and the lower die. The elements in the contact area between the specimen and the dies were made relatively fine, and the area near the initial notch was made finer, as shown in Figure 4b. A Young’s modulus of 214 GPa and a Poisson ratio of 0.3 as the conventional material properties for low-carbon steel at −196 °C [21] were used in the FEA. The strain–stress curves shown in a later figure were used for the each specimen. The yield strength, *σ_y_*, at −196 °C was 789 MPa for the SM18 specimen, 913 MPa for the SM10 specimen, and 1256 MPa for the WBR specimen. Furthermore, in the WBR specimen, the yield condition of the anisotropy in which the *y* and *z* directions yield with a stress of 0.97 times the *x* direction was included in the model as the anisotropy of the yield strength. The anisotropic strength, YS_ND_/YS_RD_ and YS_TD_/YS_RD_, was about 0.96 and 0.97, respectively. Here, YS_RD_ is the 0.2% offset yield stress parallel to the RD, and the value was 1252 MPa. The YS_RD_ = 1252 MPa was in agreement to the *σ_y_* = 1256 MPa. YS_ND_ and YS_TD_ denote the 0.2% offset yield stress parallel to the ND and TD, respectively.

## 4. Results

### 4.1. Microstructure Evolution

**Figure 5:**
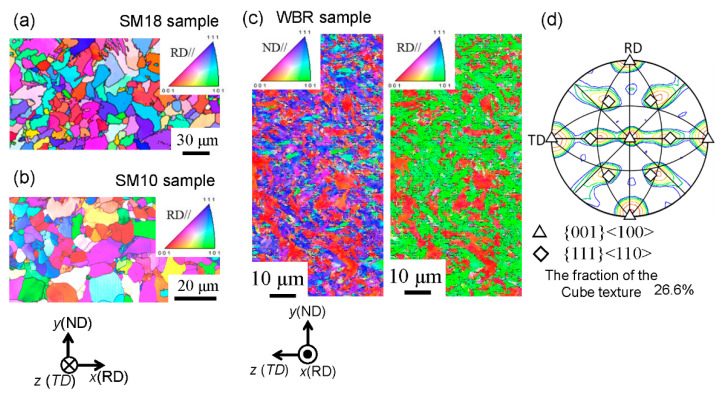
Orientation maps along the RD on the cross-sectional plane parallel to the RD at the center for (**a**) the SM18 sample and (**b**) the SM10 sample. Orientation maps along the RD and normal direction (ND) on the cross-sectional plane(**c**) normal to the RD at the center for the WBR sample, and (**d**) the (100) pole figure.

Figure 5a,b show the orientation maps along the RD for the SM18 sample and the SM10 sample. They are α structures without the strong texture observed in conventional heat-treated steel. Figure 5d,e show the orientation maps along the RD and ND for the WBR sample and the (001) pole figures corresponding to these maps. Here, the orientation maps on the cross-sectional plane parallel to the RD are given in previous paper [11]. The texture was dominated by {001}<100> cube orientations, which scarcely occurs in body-centered cubic (bcc) metals. The {111}<110> was observed as a suborientation. The fraction of the cube texture was about 26.3% under a tolerance angle of 15°.

### 4.2. Tensile Properties

**Figure 6:**
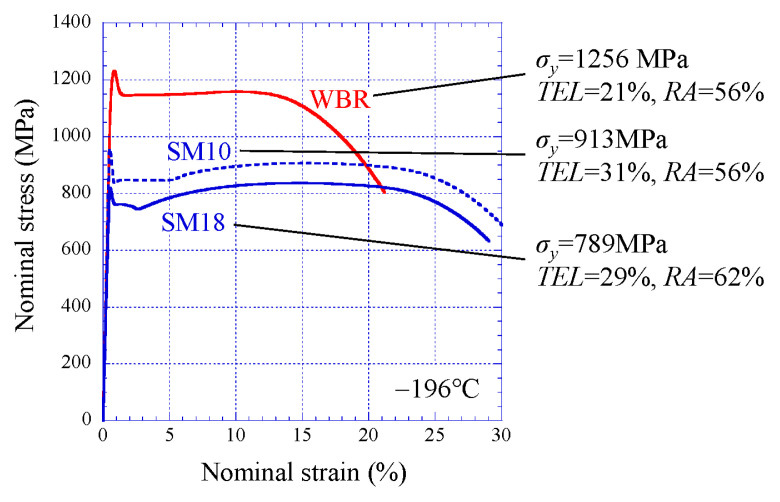
Nominal stress–nominal strain relations. Here, *σ_y_*, *TEL*, and *RA* denote yield stress, total elongation, and a reduction in area, respectively.

The nominal stress−nominal strain curves at −196 °C are shown in Figure 6. The strength of the SM10 sample was greater than that of the SM18 sample, and the total elongation, *TEL*, was large. The strength of the WBR sample significantly increased compared to the other two samples, and the *TEL* decreased. However, the reduction in area, *RA*, did not decrease significantly regardless of the improvement in strength. We found that the strength-*RA* balance of UFEG steel is superior to that in *α*-pearlite steel [10,11]. All samples exhibited a yield-drop phenomenon followed by adequate elongation. Furthermore, the SM18 and SM10 samples had a typical curve that work hardening occurs after the Lüders elongation. Sharp yield-drop phenomena are commonly seen in ultrafine-grain materials and at low temperature [22,23].

### 4.3. Three-Point Bending Properties and Fracture Stress

**Figure 7:**
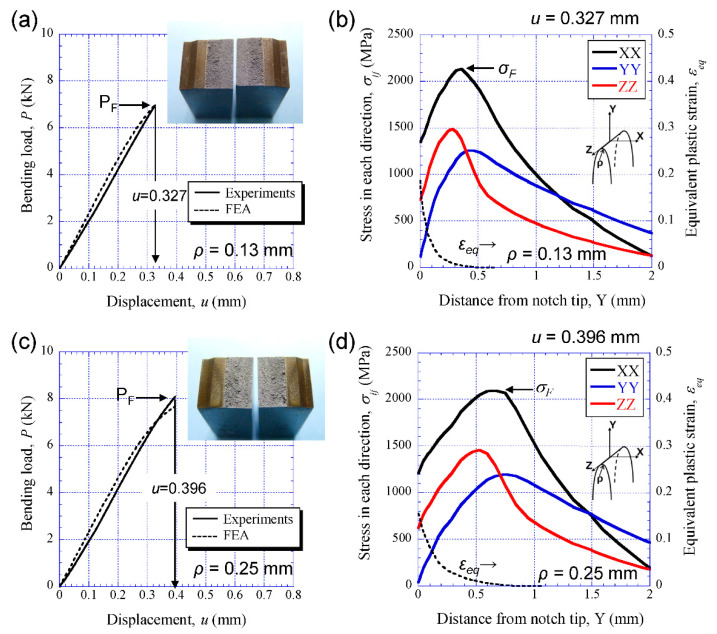
(**a**) Comparison between the *P*-*u* relations in the SM10 specimen with ρ = 0.13 mm, and the appearance of the sample. (**b**) Distributions of the stresses and the equivalent strain *ε_eq_* near the notch tip at *u* = 0.327 mm obtained via finite element analysis (FEA). (**c**) The *P*-*u* relations in the SM10 specimen with *ρ* = 0.25 mm. (**d**) Distributions of the stresses and the *ε_eq_* near the notch tip at *u* = 0.396 mm obtained via FEA. Here, *ρ* denotes the initial notch radius.

**Figure 8:**
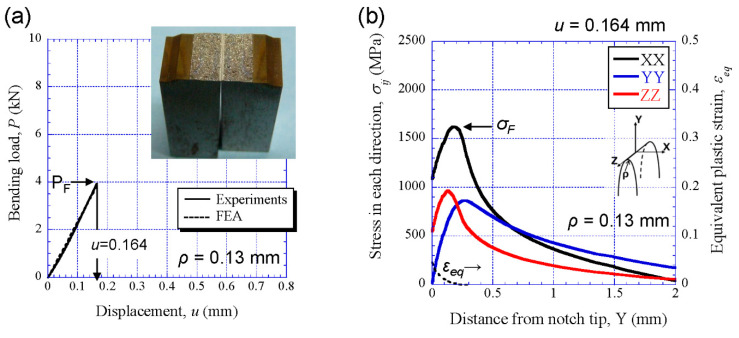
(**a**) Comparison between the *P*-*u* relations in the SM18 specimen and the appearance of the sample. (**b**) Distributions of the stresses and the equivalent strain *ε_eq_* near the initial notch tip at *u* = 0.164 mm obtained via FEA.

Figure 7a,c shows the experimental result (solid line) and FE-result (broken line) of comparing the relations of bending load *P* and displacement *u* at −196 °C for the SM10 sample, as well as the appearance of the sample after the test. The specimen immediately fractured at displacement *u* = 0.327 mm at *ρ* = 0.13 mm and *u* = 0.396 mm at *ρ* = 0.25 mm. Although the FEA result at *r* = 0.25 mm shows a slight loss of linearity just before fracture, it can be seen that the *P*-*u* relations obtained via FEA are in good agreement with the experimental results regardless of *ρ*. The stresses, *σ_XX_*, *σ_YY_*, and *σ_ZZ_*, and the equivalent plastic strain, *ε_eq_*, obtained via FEA at the *u* = 0.327 mm and *u* = 0.396 mm, respectively, are shown in Figure 7b,d. Here, the FEA results show distributions of the stresses and strain on the central cross section (the plane of symmetry in the *z* direction in Figure 4a). From Figure 7b, it is predicted that the SM10 specimen at *ρ* = 0.13 mm fractured at peak maximum stress *σ_XX(max)_*, 2134 MPa (at this time, *ε_eq_* = 0.0044). This fracture stress *σ_F_* corresponds to 2.3 times the yield strength *σ_y_* of the SM10 sample. In the SM10 specimen at *ρ* = 0.25 mm, the *σ_XX(max)_*, i.e., *σ_F_*, was 2097 MPa (at this time, *ε_eq_* = 0.0038). The location of the peak maximum stress at *ρ* = 0.25 mm is farther from the notch tip than that at *ρ* = 0.13 mm due to the effect of the notch tip. The brittle fracture stress showed almost the same value regardless of *ρ*. The results for the SM18 sample are shown in Figure 8. The SM18 specimen at *ρ* = 0.13 mm brittlely fractured at *u* = 0.164 mm, and at that time the *σ_XX(max)_* was 1624 MPa (at this time, *ε_eq_* = 0.0034). This fracture stress *σ_F_* is much lower than that of the SM10 sample.

**Figure 9:**
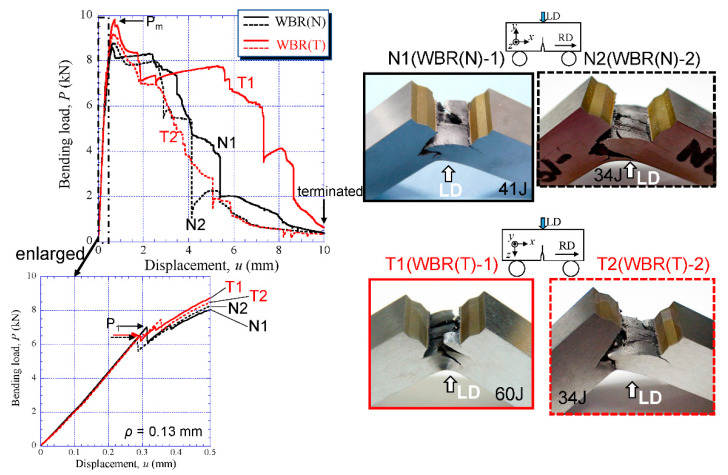
The *P*-*u* relations in the WBR(N) specimen and the WBR(T) specimen, and the appearance of the specimen.

**Figure 10:**
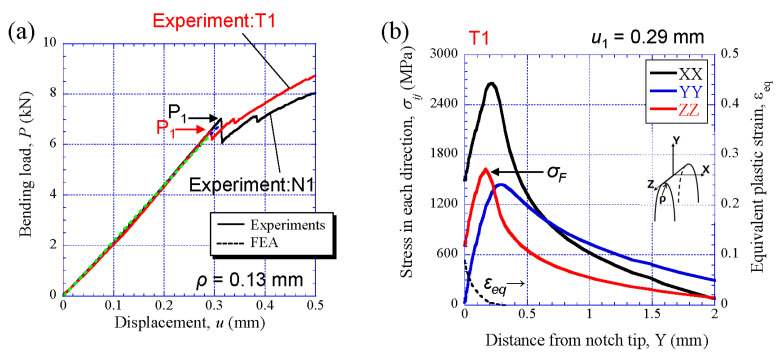
(**a**) Comparison between *P*-*u* relations obtained via experiments and FEA in the WBR(N)-1 specimen and the WBR(T)-1 specimen. (**b**) Distributions of stresses and the equivalent strain *ε_eq_* near the initial notch tip at *u*_1_ = 0.29 mm obtained via FEA for the WBR(T)-1 specimen.

The *P*-*u* relations for the WBR samples and the appearance of the sample after the test are shown in Figure 9. Here, two results performed at each notch orientation of the WBR(N) specimen (LD//ND) and the WBR(T) specimen (LD//TD) are represented. The crack advanced vertically to the LD regardless of the notch orientation, i.e., the crack branched to the longitudinal direction of the specimen. The *P* sharply dropped after it attained a maximum load, *P_m_*; beyond several load drops, *P_i_*, became almost constant, and decreased with several large load drops again thereafter. Finally, the all tests were terminated at *u* = 10 mm. A plateau region that appears after the *P_m_* results from the delamination caused by crack branching [4,11]. The *P*-*u* relations after the *P_m_* did not completely match in all specimens due to the difference of occurrence, propagation, and linkage of the microcracks on the delaminating cracks, but they showed almost the same behavior. The first load drop in the WBR(N)-1 specimen and the WBR(N)-2 specimen occurred at (*u*_1_, *P*_1_) = (0.31 mm, 7.0 kN) and (0.28 mm, 6.3 kN), respectively. In the WBR(T)-1 specimen and the WBR(T)-2 specimen, it occurred at (*u*_1_, *P*_1_) = (0.29 mm, 6.6 kN) and (0.29 mm, 6.4 kN), respectively. The results of comparing the *P*-*u* relations for the WBR(N)-1 specimen and the WBR(T)-1 specimen between the experiment (solid line) and the FEA (broken line) are shown in Figure 10a. Here, the FEA shows the results up to the displacement amount *u*_1_ in which the *P*_1_ occurred in the experiments, and it can be seen that the two are in good agreement for both specimens. Figure 10b shows the distributions of the *σ_XX_*, *σ_YY_*, *σ_ZZ_* and *ε_eq_* near the initial notch obtained via FEA at *u*_1_ = 0.29 mm for the WBR(T)-1 specimen. Although the peak maximum stress, *σ_XX(max)_*, was 2662 MPa, the stresses to pay attention to would be *σ_YY(max)_* or *σ_ZZ(max)_* normal to the X direction, because the crack branched to the longitudinal direction of the specimen. Due to *σ_YY(max)_* < *σ_ZZ(max)_*, the *σ_ZZ(max)_* = 1627 MPa (at this time, *ε_eq_* = 0.0034) might be the fracture stress that caused the first microcrack.

## 5. Discussion

### 5.1. Delaminating Crack

**Figure 11:**
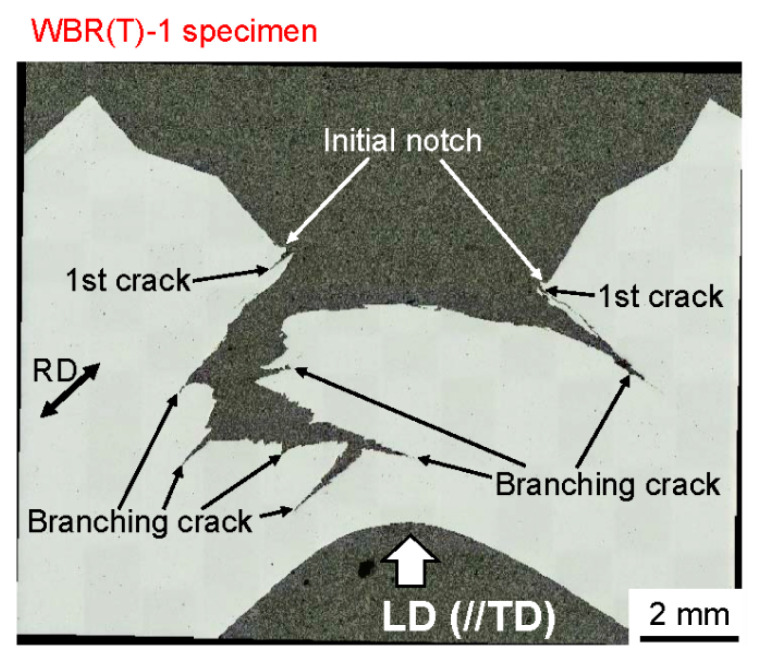
Cross-sectional image of the WBR(T)-1 specimen after the bending test.

**Figure 12:**
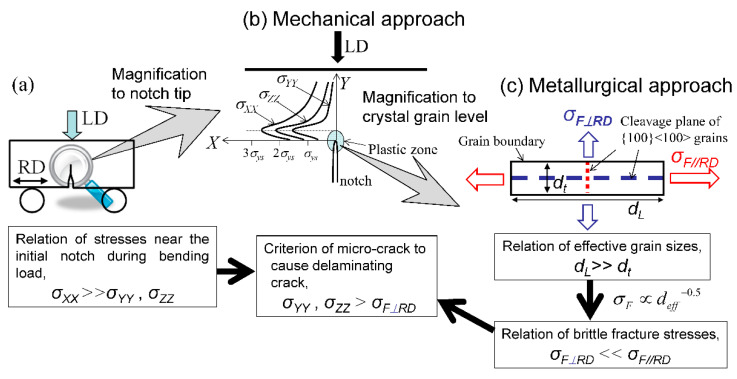
Schematic illustrations of (**a**) three-point bending test, (**b**) the stress states near the initial notch, and (**c**) the microstructure and the cleavage stresses in elongated-grain steel with a cube texture.

As seen in Figure 7 and Figure 8, in the SM10 specimen and the SM18 specimen, the crack advanced directly across the central portion of the specimen and the *σ_F_* was represented by a maximum peak stress, *σ_XX(max)_*, parallel to the longitudinal direction. In contrast, in the WBR specimen with the UFEG structure, the crack advanced vertically to the LD. The *σ_F_* was represented by a maximum peak stress, *σ_ZZ(max)_*, normal to the longitudinal direction. The initial microcrack is considered to have occurred when the first load drop appeared during the bending test. In order to verify this, the cross section of the specimen after the bending test was observed. Figure 11 shows a cross-sectional image of the WBR(T)-1 specimen after the test. Several branching cracks parallel to the RD can be observed. The branching crack near the notch tip is the first branching crack that occurred during the bending test, and it occurred approximately 0.14 mm from the notch tip. This position corresponds to the position of the maximum peak stress, *σ_ZZ(max)_*, shown in Figure 10b. The mechanism of the first branching crack formation was shown in Figure 12. In the UFEG structures with a strong cube texture, there are {100} cleavage planes along the RD and LD, as illustrated in Figure 12c. The crack//RD is mainly situated in the ferrite matrix and its grain boundaries. The bcc iron cleaves on {100} planes and the coherence length on {100} corresponds to the cleavage crack length. The coherence length on {100} for crack//RD in the elongated grains with a cube texture is much longer than that for crack⊥RD in these grains. This structure produces a condition in which the main crack can run along the longitudinal direction. The stresses near the notch tip have a relation of *σ_XX_* >> *σ_ZZ_* > *σ_YY_*, as shown in Figure 10b. Moreover, the fracture stress has a relation of *σ_F__⊥RD_* << *σ_F//RD_* from *σ_F_* ∝ *d_eff_*_−0.5_ on the basis of the Griffith theory, where *d_eff_* denotes an effective grain size for cleavage fracture. Since the brittle fracture occurs when the stress *σ_ij_* exceeds the fracture stress, a delaminating crack related to a brittle fracture induced by *σ_ZZ_*, *σ_YY_* > *σ_F__⊥__RD_* occurred before *σ_XX_* > *σ_F//RD_* or a ductile fracture. As a result, the first crack parallel to the RD near the initial notch occurred as shown in Figure 11, and the first load drop, *P*_1_, appeared (Figure 10a).

### 5.2. Brittle Fracture Stress vs. Grain Size

From the Griffith equation, the *σ_F_* is represented by

(1) $$\sigma_{F}=\sqrt{\frac{2\;E\;\gamma_{s}}{\pi \;(1-\nu^{2})}\;}\times d_{eff}{}^{-0.5}$$

Here, *E* denotes Young’s modulus, *γ_s_* is the surface energy of the fracture, and *ν* is Poisson’s ratio [24]. Actually, a shape factor related to the method of fracture test must be taken into account in Equation (1). Under this three-point bending test, the *σ_F_* is represented by the following [16,25]:

(2) $$\sigma_{F}=1.41\;\sqrt{\frac{2\;E\;\gamma_{s}}{\pi \;(1-\nu^{2})}\;}\times d_{eff}{}^{-0.5}$$

Generally, the *d_eff_* is the *α* grain size and the *σ_F_* is determined through experiments and FEA. In the case of the WBR samples, the *d_eff_* (in this case, *d_effL_*) for the fracture stress *σ_F_*_⊥*RD*_, as shown in Figure 12c, corresponds to the average grain size, *d_L_*, in a longitudinal direction. It is difficult to accurately measure the size of *d_L_* from EBSP maps and the SEM images of the delaminating fracture surface, but the *d_effL_* can be calculated from the results of the equiaxed grain sample and *σ_F_*_⊥*RD*_. Assuming the *γ_s_*, *E*, and *ν* in the SM18 sample and the WBR sample are the same, the effective grain size, *d_effL_*_(*WBR*)_, of the WBR sample is expressed by

(3) $$\sigma_{F(SM18)}\sqrt{d_{eff(SM18)}}=\sigma_{F⊥RD(WBR)}\sqrt{d_{effL(WBR)}}\;\to \;d_{effL(WBR)}=d_{eff(SM18)}{\left(\frac{\sigma_{F(SM18)}}{\sigma_{F⊥RD(WBR)}}\right)}^{2}$$

Using *d_eff(SM18)_* = 18.0 μm, *σ_F_*_(*SM18*)_ = 1624 MPa, and *σ_F_*_⊥*RD(WBR)*_ = 1627 MPa, the *d_effL_*_(*WBR*)_ is estimated to be 17.9 μm. The fracture stress, *σ_F_*_//*RD*(*WBR*)_, parallel to the RD is described by

(4) $$\sigma_{F⊥RD(WBR)}\sqrt{d_{effL(WBR)}}=\sigma_{F//RD(WBR)}\sqrt{d_{efft(WBR)}}\;\to \;\sigma_{F//RD(WBR)}=\sigma_{F⊥RD(WBR)}\sqrt{\frac{d_{effL(WBR)}}{d_{efft(WBR)}}}$$

**Figure 13:**
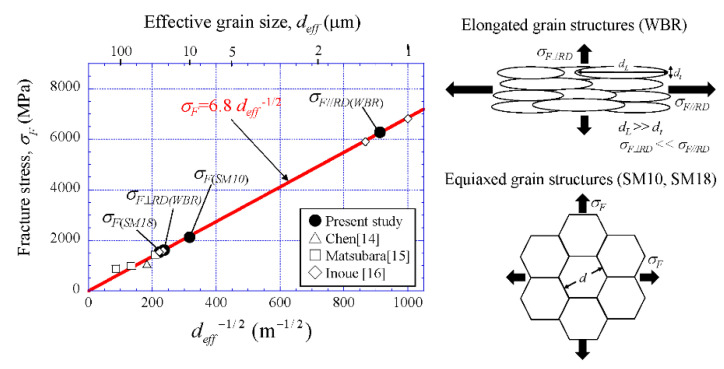
Fracture stress vs. effective grain size.

The *σ_F_*_//*RD*(*WBR*)_ is 6284 MPa using *d_efft_*_(*WBR*)_ = 1.2 μm. That is, UFG steel with a grain size of 1.2 μm is a very high fracture stress of about 6.3 GP. Figure 13 shows the fracture stresses plotted as a function of the inverse square root of *d_eff_*, together thewith the data shown in past literature [14,15,16]. The effective surface energy is estimated to be 158 J m^−2^ based on this linear relation and Equation (2). Here, *E* = 214 GPa and *ν* = 0.30 were used in this relation [21]. This value is included in the ranges of 90–190 J m^−2^ for C-Mn steels with *α*-pearlite microstructure [26].

### 5.3. Condition for Brittle Fracture

The *σ_y_* increases with *k* × *d_α_*^−1/2^ under the Hall–Petch relationship. In the previous study [11], the coefficient *k* was 0.6 MPa·m^−1/2^ regardless of temperature in low-carbon steel, and this is consistent with the result reported by other researchers [8,23]. The slope, 6.8, in the *σ_F_* − *d_eff_*^−1/2^ relation shown in Figure 13 is 10 times larger than the *k*. This indicates that the DBTT is significantly improved by grain refinement. To quantitatively verify this, the conditions of brittle fracture in low-carbon steel under the present bending test and Charpy impact test were examined through the results of Charpy test obtained in previous papers [11,20] and the results of the present study for the SM18 sample.

**Figure 14:**
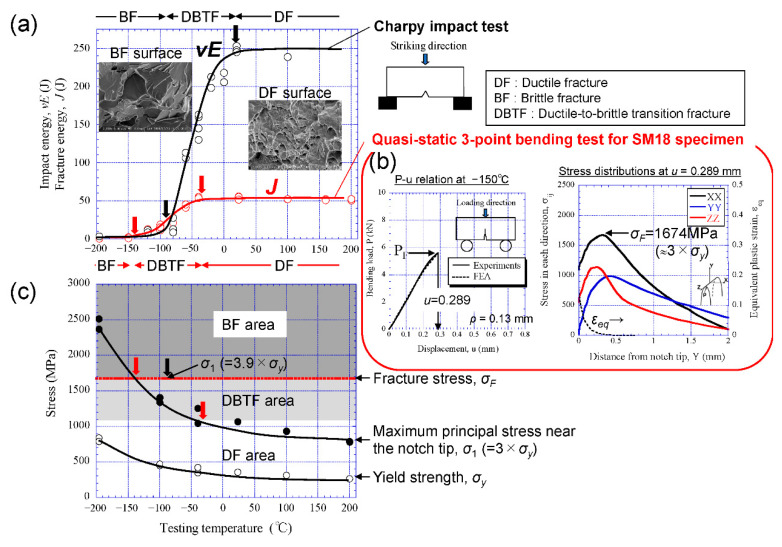
Variations in the (**a**) fracture energy and (**c**) stress with temperature for the SM18 sample, where (**a**) *vE* denotes the Charpy impact absorbed energy [11] and *J* denotes the fracture energy obtained in the three-point bending test shown in Figure 2a [20], and (**c**) *σ*_1_ denotes the maximum tensile stress near a notch tip and is about 3 times *σ_y_* under the present bending test. (**b**) Comparison between the *P*-*u* relations at −150 °C obtained via experiments and FEA and distributions of the stresses and the equivalent strain *ε_eq_* near the initial notch tip at *u* = 0.289 mm obtained via FEA.

Figure 14a shows variations in the Charpy absorbed impact energy, *vE* (Figure 9 in [11]), and fracture energy, *J* (Figure 8b in [20]), with the temperature for the SM18 sample. Here, the *vE* curve was obtained from the Charpy impact tests with full-size V-notched specimens under a 500 J capacity [11], and the *J* denotes the area under the *P* − *u* curve, as shown in Figure 8a. The sample exhibits a typical energy-transition curve, in which the *vE* and *J* decrease with decreasing temperature. In the three-point bending test, the *J* starts to increase from below about −140 °C. That is, the sample transfers from brittle fracture to ductile fracture at that point. The *P* − *u* relation at −150 °C and the stress distributions near the initial tip at *u* = 0.289 mm, in which the sample exhibited catastrophic fracture, are shown in Figure 14b. These results were obtained using the same approach as for the result at −196 °C (Figure 8a). The *σ_F_* at −150 °C was 1674 MPa, and this value is almost in agreement with the *σ_F_* at −196 °C, shown in Figure 8b. The *σ_F_* corresponds to about 3 times the *σ_y_* (=560 MPa) at −150 °C. On the basis of the Yoffee diagram [12], the DBTT occurs when the maximum tensile stress near the notch/crack tip, *σ*_1_, exceeds the *σ_F_*. The *σ*_1_ has to be calculated via FEA. Since the *σ*_1_ is about 3 times the *σ_y_* in the present bending test, a relation among the *σ_F_*, the *σ*_1_ and temperature is shown in Figure 14c. The *σ_F_* is independent of temperature. Hence, the *σ*_1_ that increases with decreasing temperature exceeds the *σ_F_* at about −140 °C. This indicates that the sample exhibits complete brittle fracture below −140 °C, complete ductile fracture above −30 °C, and ductile-to-brittle transition fracture (DBTF) between those temperatures. In contrast, in the Charpy impact test, although it is difficult to precisely calculate the *σ*_1_ near the notch during the impact test due to high-speed deformation and heat generation, the *σ*_1_ increases as the strain rate increases. According to the FEA results of Tanguy et al. [27] and Takashima and Minami [28], the *σ*_1_ is about 3.5–4 times the *σ_y_*. Since the *σ_F_* is independent of the strain rate, complete brittle fracture can be predicted to appear even at temperatures above −140 °C obtained in the three-point bending test. The *vE* curve in Figure 14a,c proves this fact. The sample shows complete brittle fracture at below −90 °C, indicated by a black arrow. The *σ_y_* at −90 °C is about 430 MPa, and the *σ_F_* = 1674 MPa corresponds to about 3.9 times the *σ_y_*. This is consistent with the FEA results of *σ*_1_ ≈ (3.5–4) *σ_y_* [27,28].

**Figure 15:**
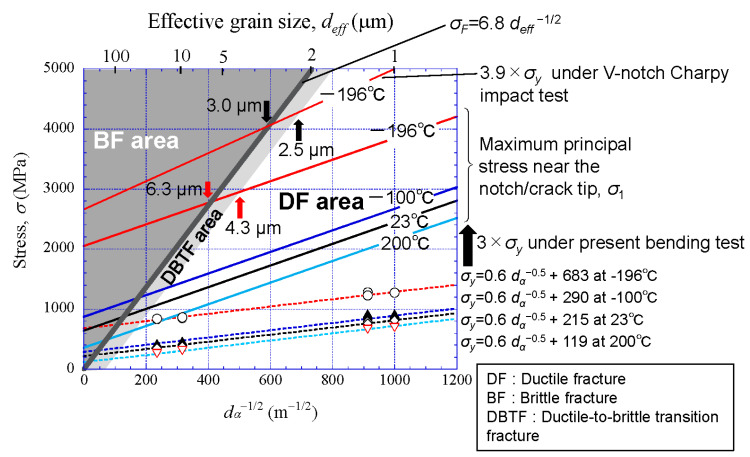
Conditions for brittle fracture in low-carbon steel on the basis of a comparison of the grain-size dependence of maximum tensile stress and brittle fracture stress.

From Figure 13 and the Hall–Petch relationship, we can quantitatively show the superiority of the *α* grain size for brittle fracture. Figure 15 shows a comparison of the grain-size dependence of maximum tensile stress and brittle fracture stress. This result indicates that a low-carbon steel with a *α* grain size of 6.3 µm or more exhibits complete brittle fracture at −196 °C, and one with a *α* grain size of 4.3 µm or less exhibits complete ductile fracture even at −196 °C. When the *ρ* of the initial notch or ligament length in the test specimen is small or when a fracture test is conducted under a high strain rate, these grains shift toward a finer size. In the Charpy impact test, since the maximum tensile stress near the V-notch in a specimen is assumed to be 3.9 times the *σ_y_*, it is predicted via the stress–*d_α_*^−1/2^ map shown in Figure 15 that a low-carbon steel with a *α* grain size of 3.0 µm or more exhibits complete brittle fracture at −196 °C, and a steel with a *α* grain size of 2.5 µm or less exhibits complete ductile fracture. In the general fracture test, the maximum tensile stress near the crack or notch tip is of the order of (2–4) *σ_y_* [14,27,28,29,30]. If a ferrite grain can be ultrarefined to 1 μm, the material would exhibit complete ductile fracture even under liquid nitrogen temperature. Through the maps shown in Figure 14c and Figure 15, we can estimate the grain size and temperature at which ductile-to-brittle transition fracture occurs.

## 6. Conclusions

The UFEG low-carbon steel with atransverse grain size of 1.2 μm dominated by a cube texture was created via multipass warm biaxial rolling. Two low-carbon steels with a ferrite (*α*)-pearlite structure with grain size 10 μm and 18 μm were also prepared via conventional rolling and heat treatment. These samples were studied from the viewpoint of the toughness criterion, ductile-to-brittle transition fracture, and brittle fracture stress through mechanical tests and FEA. The main results are summarized as follows.

(1) The brittle fracture stress (*σ_F_*) was estimated through the three-point bending test and FEA. The *σ_F_* for low-carbon steels with a grain size of 18 μm, 10 μm, and 1.2 μm was about 1674 MPa, 2134 MPa, and 6284 MPa, respectively.

(2) The *σ_F_* and ferrite grain size (*d_α_*) had a relation of *σ_F_* = 6.8 *d_α_*^−1/2^. The slope, 6.8, is 10 times larger than in the Hall–Petch relationship. This indicates that grain refinement significantly improves the DBTT.

(3) The conditions of *α* grain size and temperature that cause a ductile-to-brittle fracture in low-carbon steel were clarified. We can understand them via the stress −*d_α_*^−1/2^ map. The superiority of the *α* grain size for brittle fracture was quantitatively shown.

## Data Availability

Data sharing is not applicable to this article.
